# Two Cases of *Neisseria meningitidis* Proctitis in HIV-Positive Men Who Have Sex with Men

**DOI:** 10.3201/eid2303.161039

**Published:** 2017-03

**Authors:** José Gutierrez-Fernandez, Verónica Medina, Carmen Hidalgo-Tenorio, Raquel Abad

**Affiliations:** University of Granada, Granada, Spain (J. Gutierrez-Fernandez);; University Hospital Virgen de las Nieves, Granada (J. Gutierrez-Fernandez, C. Hidalgo-Tenorio);; Instituto de Salud Carlos III, Majadahonda, Madrid, Spain (V. Medina, R. Abad)

**Keywords:** proctitis, Neisseria meningitidis, bacteria, men who have sex with men, HIV, HIV/AIDS and other retroviruses, sexually transmitted infections, molecular characterization

## Abstract

We report 2 cases from Spain of infectious proctitis caused by *Neisseria meningitidis* in HIV-positive men who have sex with men. Genetic characterization of the isolates showed that they are unusual strains not found in other more frequent meningococcal locations. This finding suggests an association between specific strains and anogenital tract colonization.

Pathogens that cause proctitis include *Neisseria gonorrhoeae*, *Chlamydia trachomatis*, *Treponema pallidum*, and herpes simplex virus (*1*). We report 2 cases from Spain of proctitis caused by *Neisseria meningitidis*, a pathogen less frequently detected.

The first case-patient was a 32-year-old HIV-positive man who had proctalgia and purulent anal and urethral discharges. He reported having unprotected sex with other men.

The second case-patient was a 49-year HIV-positive man who had a purulent discharge, pain, and anal tenesmus. He reported having unprotected anal sex with other men and having previously diagnosed sexually transmitted infections.

Both patients were given a diagnosis of probable infectious proctitis. Rectal exudates samples were collected for detection of infectious agents. The first case-patient was given ceftriaxone (1 g, single intramuscular dose) and azithromycin (1.5 g, single oral dose). The second case-patient was given ceftriaxone (250 mg, single intramuscular dose). Both patients showed clinical improvement.

Routine screening for *Mycoplasma* spp. and nucleic acid amplification for *N. gonorrhoeae* and *C. trachomatis* yielded negative results. We isolated gram-negative diplococci from both patients on modified Martin-Lewis agar (Becton Dickinson, Franklin Lakes, NJ, USA) and identified these diplococci as *N. meningitidis* serogroup B by using mass spectrometry (Biotyper System; Bruker, Billerica, MA, USA) and standard biochemical tests (Vitek II; bioMérieux, Marcy l’Etoile, France). 

We determined MICs by using Etest (bioMérieux). MICs were 0.047 mg/L for the isolate from first patient and 0.19 mg/L for the isolate from the second patient for tetracycline; 0.125 and 0.016 mg/L for cefotaxime; 0.004 and 0.006 mg/L for ciprofloxacin, 0.75 and 2 mg/L for azithromycin; 0.047 and 0.25 mg/L for penicillin; 0.38 and 1.5 mg/L for ampicillin; <0.002 and <0.002 mg/L for ceftriaxone; and 0.064 and 0.094 mg/L for rifampin.

The isolates were sent to the National Reference Laboratory in Madrid, Spain, for confirmation of identification and characterization. Molecular characterization included genotyping by sequencing variable regions of the PorA protein gene, (*2*), multilocus sequence typing (MLST) (*3*), and FetA protein variable region gene characterization (*4*).

The isolates were identified by using slide agglutination with specific polyclonal antibodies as being *N. meningitidis* serogroup B. The isolate from first case-patient was characterized as genosubtype P1.22,14-13 (PorA VR1:22, VR2:14-13), FetA type F5–7 (FetA VR:5-7), sequence type (ST) 10866, with a clonal complex (CC) not assigned (NA) (i.e., a B:P1.22,14-13:F5-7:ST10866 CCNA strain). The isolate from second case-patient was characterized as B:P1.17-6,23-6:F3-36:ST3469 (CC4821).

We did not find similar isolates at the National Reference Laboratory for patients with invasive meningococcal disease (IMD), healthy carriers, or persons with urogenital infections. Only 4 strains in the same CC as that for the isolate from the second case-patient were found in the MLST database (http://pubmlst.org/neisseria/http://pubmlst.org/neisseria/); these isolates had a similar genosubtype and FetA type, but only 1 isolate, obtained from a carrier in Australia in 2014, had the same ST.

B:22,14 strains are found more frequently in IMD patients and healthy carriers. However, more strains are ST213CC, which is the second most prevalent CC in Spain. We found 2 strains with the same ST as the strain from the first case-patient in the MLST database. Both of these strains were isolated from men who had sex with men in Brighton, UK, 1 isolated in 2013 from a urethral swab specimen (22-4,14-13:F5-7:ST10866) and the other isolated in 2012 from rectal swab specimen (22,14-13:F5-7:ST10866).

The natural habitat of *N. meningitidis* is the human nasopharynx. However, it occasionally enters the bloodstream and causes IMD characterized by meningitis or septicemia (*5*). *N. meningitidis* has been isolated from the urethra, cervix, and anal canal and has been reported as a cause of anogenital infection (*6**–**8*). Orogenital contact is the most probable route of *N. meningitidis* transmission from nasopharynx to urogenital tract and anal canal (*8*), which has been associated mainly with heterosexual patients (*8*). The pathogenic role for rectal infection with *N. meningitidis* is unclear because of a low frequency of symptomatic infected patients. However, histopathologic changes have been reported in rectal mucous of patients infected with *N. meningitidis* (*9*).

*N. meningitidis* is highly variable because it can naturally undergo transformation, which leads to changes in virulence and transmissibility and suggests that new variants could emerge that have increased fitness for alternative/novel niches (*10*). This suggestion could be useful in identifying *N. meningitidis* strains with ST10866, which have been isolated from patients with anogenital infections and might be one of those variants. Whether HIV infection, with its associated immune problems, favors colonization with other microorganisms adapted to different ecologic niches has not been resolved.

Although an increased prevalence of meningococcal anogenital infections has been reported (*6*–*8*), the incidence of these infections is probably still underestimated because *N. meningitidis* might be the etiologic agent in patients with gonococcal-like urethritis and proctitis. This underestimation could be caused, in part, by use of PCR as the only diagnostic method. Thus, culture is still needed for isolating strains and determining their antimicrobial drug resistance. Monitoring the incidence of meningococcus reproductive tract infections and genetic characterization are necessary to determine the magnitude and clinical role of these infections.
